# Supplementary material for: A Randomized Phase 2/3 Study of Ensitrelvir, a Novel Oral SARS-CoV-2 3C-Like Protease Inhibitor, in Japanese Patients with Mild-to-Moderate COVID-19 or Asymptomatic SARS-CoV-2 Infection: Results of the Phase 2a Part
Source: Antimicrob Agents Chemother. 2022 Sep 13;66(10):e00697-22. doi: 10.1128/aac.00697-22 (PMC9578433; doi:10.1128/aac.00697-22)
Supplement: Supplemental file 1 — Supplemental material. Download aac.00697-22-s0001.pdf, PDF file, 0.9 MB [file aac.00697-22-s0001.pdf]

Supplemental material

FIG S1 Proportion of patients with positive SARS-CoV-2 viral titer (mITT population)

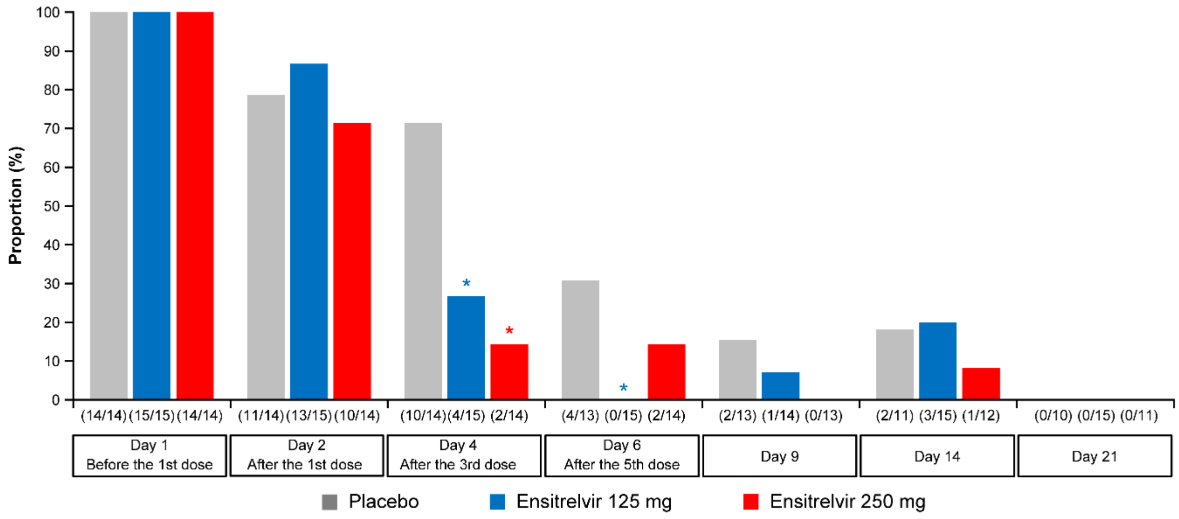

**P* < 0.05 versus placebo.

mITT, modified intention-to-treat; SARS-CoV-2, severe acute respiratory syndrome coronavirus 2.

FIG S2 Mean absolute values of each of the 14 COVID-19 symptom scores (ITT population)

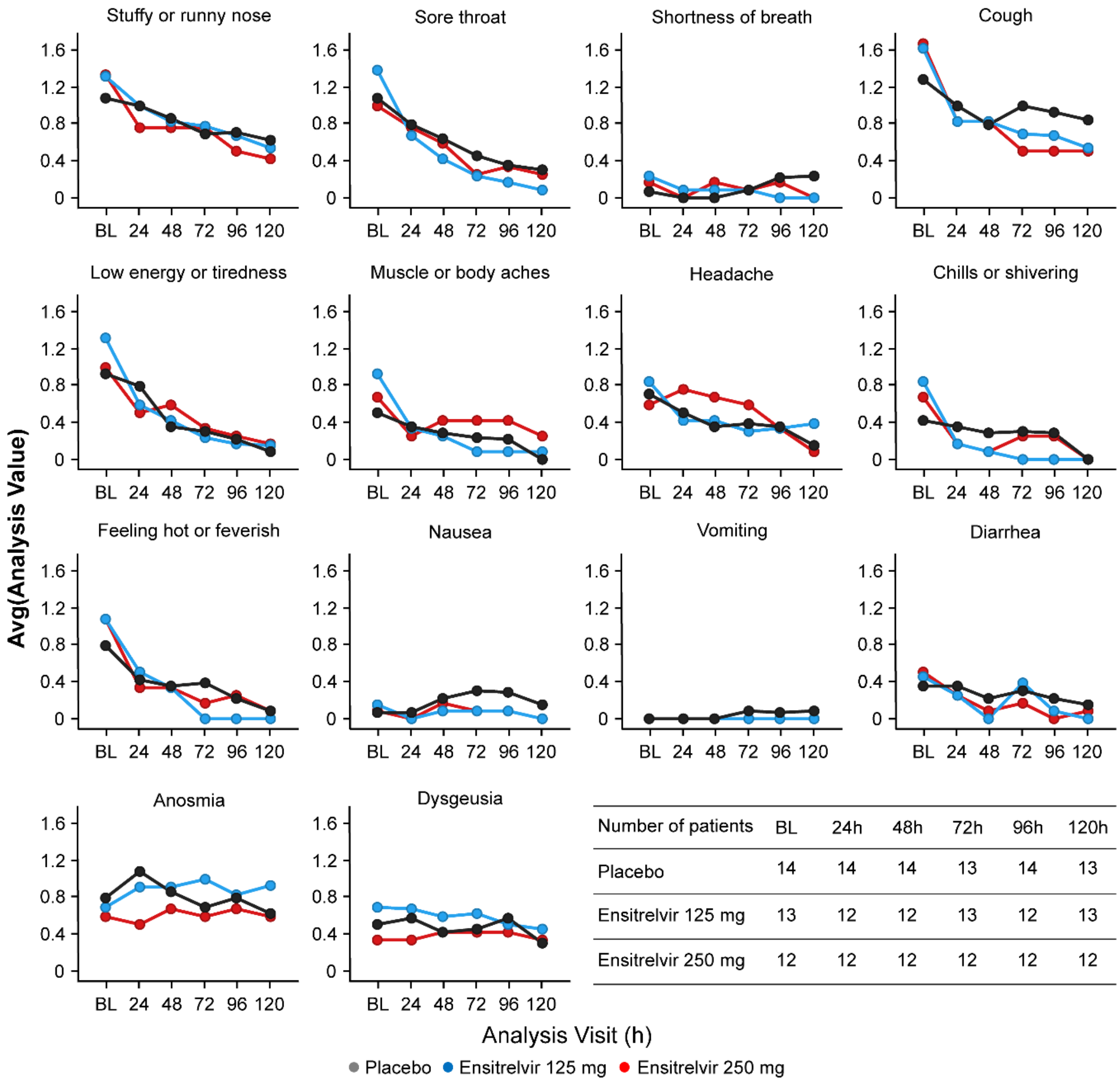

Number of patients	BL	24h	48h	72h	96h	120h
Placebo	14	14	14	13	14	13
Ensitrelvir 125 mg	13	12	12	13	12	13
Ensitrelvir 250 mg	12	12	12	12	12	12

Avg, average; BL, baseline; COVID-19, coronavirus disease 2019; h, hours; ITT, intention-to-treat.

FIG S3 Mean change from baseline in each of the 14 COVID-19 symptom scores (ITT population)

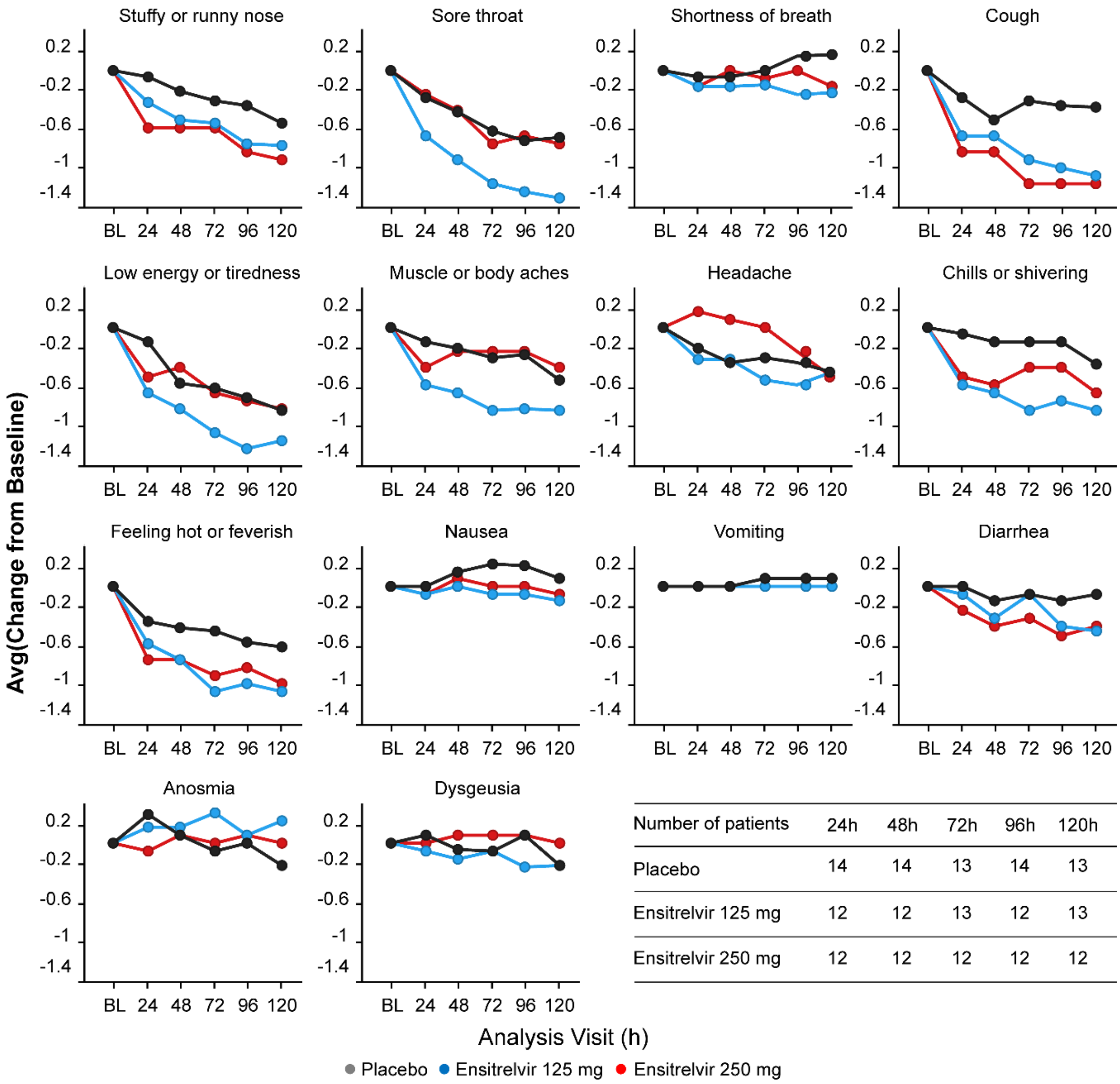

Number of patients	24h	48h	72h	96h	120h
Placebo	14	14	13	14	13
Ensitrelvir 125 mg	12	12	13	12	13
Ensitrelvir 250 mg	12	12	12	12	12

Avg, average; BL, baseline; COVID-19, coronavirus disease 2019; h, hours; ITT, intention-to-treat.

FIG S4 HDL cholesterol and blood triglyceride levels as (A) absolute values and (B) change from baseline (safety analysis set)

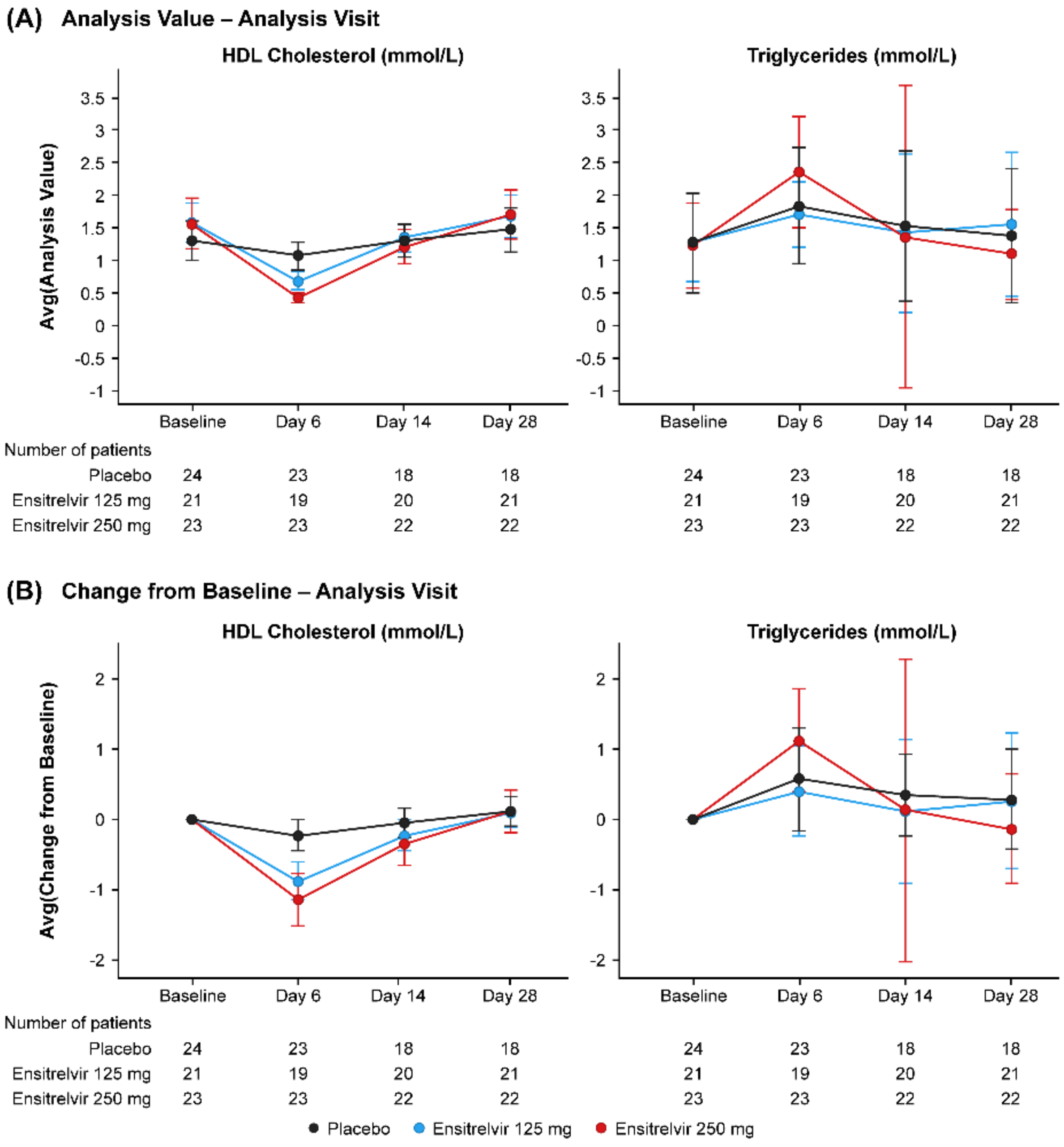

Data are presented as mean ± SD.

HDL, high density lipoprotein; SD, standard deviation.

FIG S5 Total bilirubin and iron levels as (A) absolute values and (B) change from baseline (safety analysis set)

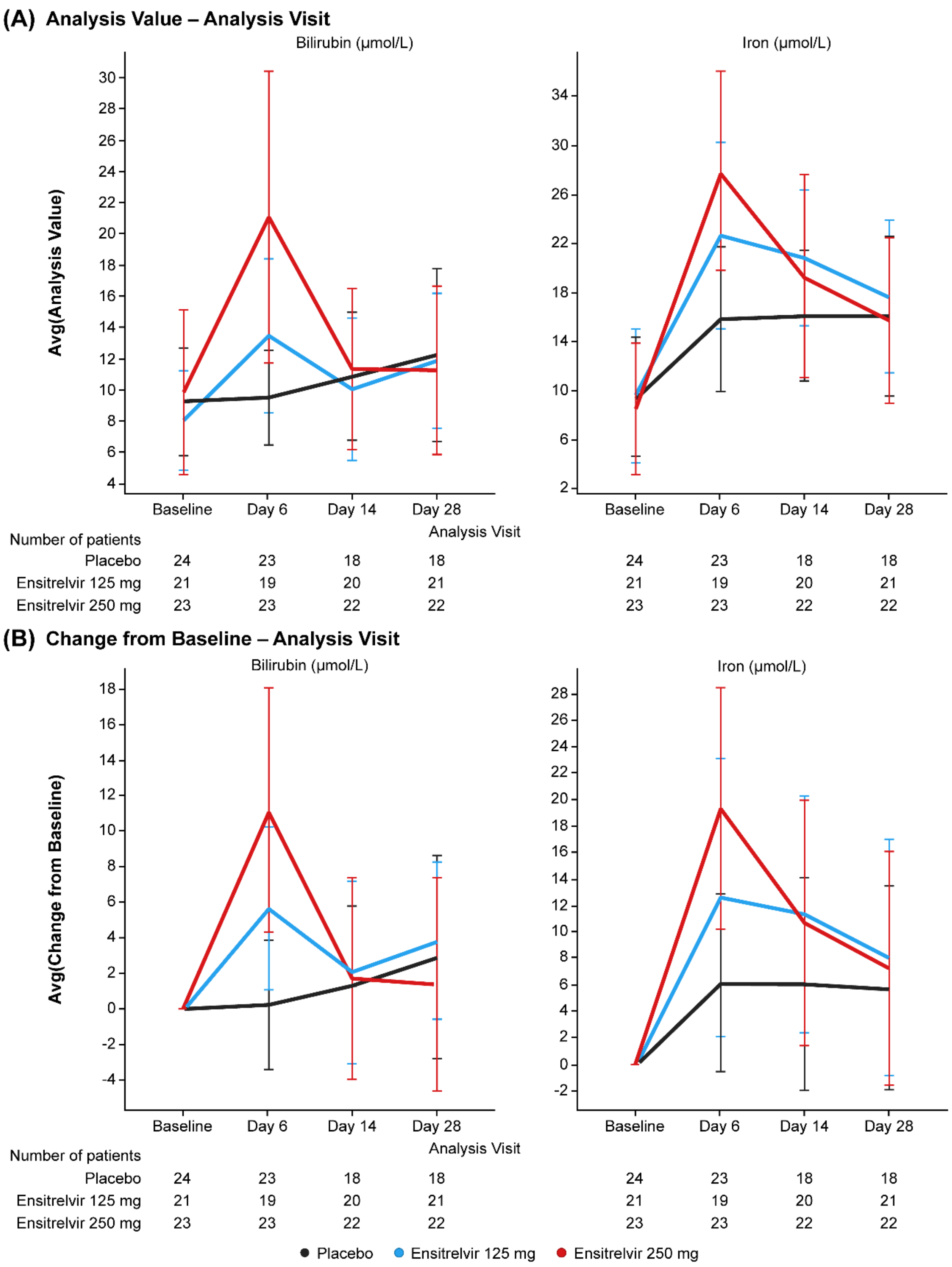

Data are presented as mean ± SD.

SD, standard deviation.

TABLE S1 Proportion of patients with mild-to-moderate COVID-19 who had disease exacerbation (≥3 in the 8-point ordinal scale) after treatment initiation (ITT population)

**Patients**	**Ensitrelvir 125 mg (N=13)**	**Ensitrelvir 250 mg (N=12)**	**Placebo (N=14)**
Patients with exacerbation, n/N (%)			
Overall	0/13 (0.0)	0/12 (0.0)	2/14 (14.3)
Unvaccinated	0/2 (0.0)	0/2 (0.0)	2/4 (50.0)
Vaccinated	0/11 (0.0)	0/10 (0.0)	0/10 (0.0)

Data are derived from mild-to-moderate patients in the ITT population.

COVID-19, coronavirus disease 2019; ITT, intention-to-treat.

TABLE S2 Questionnaire for the COVID-19 symptom scores

**Questionnaire item**	**Response options and scoring**
Respiratory symptoms	None=0
1. Stuffy or runny nose[a]	Mild=1
2. Sore throat[a]	Moderate=2
3. Shortness of breath (difficulty breathing)[a]	Severe=3
4. Cough[a]	
Systemic symptoms	
5. Low energy or tiredness[a]	
6. Muscle or body aches[a]	
7. Headache[a]	
8. Chills or shivering[a]	
9. Feeling hot or feverish[a]	
Digestive symptoms	
10. Nausea (feeling like you wanted to throw up)[a]	
11. Vomiting (throwing up)	
12. Diarrhea (loose or watery stools)	
Sensation disturbance	Same as usual=0
13. Rate your sense of smell in the last 24 hours[b]	Less than usual=1
14. Rate your sense of taste in the last 24 hours[b]	No sense of smell/taste=2

[a]Patients were asked to rate the severity of their symptoms at their worst over the last 24 hours.

[b]Not used to calculate the total score of the 12 COVID-19 symptoms.

COVID-19, coronavirus disease 2019.

TABLE S3 The 8-point ordinal scale for patients' conditions

**Descriptor**	**Score**
Asymptomatic	0
Symptomatic, no limitation of activities	1
Symptomatic, limitation of activities	2
Hospitalized, no oxygen therapy	3
Hospitalized, with oxygen therapy (<5 L/min)	4
Hospitalized, with oxygen therapy (≥5 L/min)	5
Hospitalized, with ventilation	6
Death	7
